# Liver cancer in Hidalgo State, Mexico: analysis of the status, risk factors and regional public health policy requirements: a cross-sectional correlational study

**DOI:** 10.1590/1516-3180.2021.0601.R1.121121

**Published:** 2022-06-27

**Authors:** Rosa Isela Barrera-Cortés, Erika Elizabeth Rodriguez-Torres, Enrique Vázquez-Mendoza, Jesús Carlos Ruvalcaba-Ledezma, Luis Enrique Soria-Jasso, Mario Isidoro Ortiz, Eduardo Fernández-Martínez

**Affiliations:** I MD, MPH. Family Medicine Specialty Student, Department of Medicine, School of Health Sciences, Universidad Autónoma del Estado de Hidalgo (UAEH), Pachuca, Hidalgo, Mexico.; II BSc, MSc, PhD. Full Professor, Department of Mathematics and Physics, School of Basic Sciences and Engineering, Universidad Autónoma del Estado de Hidalgo (UAEH), Pachuca, Hidalgo, Mexico.; III BSc, MSc. Doctoral Student, Department of Mathematics and Physics, School of Basic Sciences and Engineering, Universidad Autónoma del Estado de Hidalgo (UAEH), Pachuca, Hidalgo, Mexico.; IV MD, PhD. Full Professor, Department of Medicine, School of Health Sciences, Universidad Autónoma del Estado de Hidalgo (UAEH), Pachuca, Hidalgo, Mexico.; V MD, PhD. Full Professor, Department of Medicine, School of Health Sciences, Universidad Autónoma del Estado de Hidalgo (UAEH), Pachuca, Hidalgo, Mexico.; VI MD, MSc, PhD. Full Professor, Department of Medicine, School of Health Sciences, Universidad Autónoma del Estado de Hidalgo (UAEH), Pachuca, Hidalgo, Mexico.; VII BSc, MSc, PhD. Full Professor, Department of Medicine, School of Health Sciences, Universidad Autónoma del Estado de Hidalgo (UAEH), Pachuca, Hidalgo, Mexico.

**Keywords:** Alcoholism, Health policy, Liver neoplasms, Regional medical programs, Risk factors, Public health, Cirrhosis, Hepatocellular carcinoma, Marginalization, Non-alcoholic steatohepatitis, Regional strategies

## Abstract

**BACKGROUND:**

In Latin America, liver cancer is one of the top causes of cancer mortality. It is the fifth most common cause of death among malignant tumors in Mexico and is the leading cause in Hidalgo State (43.8% of the population living in poverty).

**OBJECTIVE:**

To conduct a correlational analysis on the main risk factors for liver cancer in Hidalgo State, Mexico, including municipal disaggregation and comparison with the national level.

**DESIGN AND SETTING:**

Cross-sectional, correlational, descriptive and comparative epidemiological study using Mexican governmental databases covering 1990-2019.

**METHODS:**

A comprehensive review of the databases of the General Directorate of Health Information (DGIS) was performed to analyze official death figures, hospital discharges and national and municipal population projections, using specific search criteria defined in the Global Burden of Disease classification, based on the risk factors for liver cancer.

**RESULTS:**

Liver cancer rates showed an evident rise in Hidalgo (183%), moving from 21^st^ place in Mexico in 1990 to 9^th^ place in 2019. This increase was correlated with alcoholism. An increasing trend for liver cancer deaths, of 133.89%, is projected for 2030. Females and the population over 60 years of age are more affected. There are some critical regions with liver cancer death rates twice the national rate or more.

**CONCLUSION:**

Targeted effective public health strategies should be structured by identifying, characterizing and regionalizing critical marginalized municipalities that are vulnerable to alcoholism and other risk factors for liver cancer. This approach may be helpful for other states in Mexico or similar countries.

## INTRODUCTION

There has been an increase in the global incidence of liver cancer, which became the sixth most common cancer and the third leading cause of cancer death in the world in 2020.^1^ This has also raised interest in studying its clinical and epidemiological aspects in Latin America.^2^ ^,^ ^3^ ^,^ ^4^ Hepatocellular carcinoma (HCC) is the most frequent form of liver cancer, accounting for 75% to 85% of primary hepatic neoplasms.^1^ It is also between the second and fourth most important cause of cancer-related mortality worldwide and is the leading cause of cancer among patients with cirrhosis.^2^ ^,^ ^3^ ^,^ ^5^ HCC occurs more frequently in men than in women, and about one million new cases are diagnosed every year.^3^ ^,^ ^6^ ^,^ ^7^

In Mexico, complications from liver cirrhosis have remained the third most common cause of death over the last decades, and the future trend is worrying. The incidence of HCC continues to rise in Mexico, especially affecting older people in the sixth decade of life, and the mortality rate used to be higher among males. The most common underlying chronic liver diseases that cause HCC in the Mexican population are alcoholic liver disease (ALD) and hepatitis C virus (HCV). However, non-alcoholic steatohepatitis (NASH) is increasingly a cause of HCC: the incidence of this disease is rising at an alarming rate because of complications from metabolic syndrome (MetS), which is present in a high proportion of Mexicans.^8^ ^,^ ^9^ Although the epidemiological characteristics of HCC in Mexico are similar to those in other Latin American countries, such as Argentina, Brazil and Colombia,^4^ comprehensive information concerning the diagnosis, treatment and surveillance of HCC in Mexico is scarce. Better information might improve early detection and thus decrease morbidity and mortality among HCC patients.^7^ ^,^ ^8^ ^,^ ^10^

Hidalgo State, Mexico, had an economic complexity index (ECI) of -0.68 in 2021 and ranked 21^st^ among the country’s 32 states, while the overall ECI for Mexico was 1.31 in 2020. Over 43.8% of Hidalgo’s population live in poverty, and the average illiteracy rate in 2020 was 6.6%. The average social marginalization of Hidalgo in 2015, according to the GINI index, was 0.421. The medical attention options and healthcare coverages for inhabitants of Hidalgo are hospitals and care clinics belonging to the Ministry of Health (Secretaría de Salud, SS) and the Mexican Institute of Social Security (Instituto Mexicano del Seguro Social, IMSS) with coverages of 43.3% and 20.5%, respectively.^11^

In Hidalgo, recent epidemiological data on cancer from the General Directorate of Health Information (DGIS) and the Ministry of Health (Secretaría de Salud, SS) of Mexico have revealed that HCC is the leading cause of death in this state, and this has attracted attention as a critical public health problem. In this state, the percentage of deaths caused by malignant liver tumors (MLTs) increased alarmingly from 1990 to 2013, by 152%. This contrasted with the national increase of 97.5% over the same period. The liver cancer mortality rate in Hidalgo almost doubled in 23 years, from 3.1 to 6.1 deaths per 100,000 inhabitants, while this rate was 1.6 deaths per 100,000 inhabitants in Mexico. Importantly, this cause of mortality in Hidalgo is higher than the national average.

## OBJECTIVE

The aims of this study were to conduct a correlational and descriptive analysis on the main risk factors for liver cancer in Hidalgo State (hepatic fibrosis/cirrhosis, ALD and HCV), including municipal disaggregation and comparison with the national level; and to study trends of liver cancer deaths and projections to 2030, using data covering the period from 1990-2019.

## METHODS

### Study design

This study was designed as a correlational, descriptive, cross-sectional and comparative epidemiological study. This work did not require institutional review board (IRB) approval, given that it used anonymized and publicly available data.

### Data sources

A comprehensive review of the databases of the DGIS was performed using specific search criteria for the analysis. The items were: a) deaths, according to definitive official figures for the period from 1979 to 2019 that were obtained from the National Institute of Statistics, Geography and Informatics (INEGI) and the SS of Mexico; b) hospital discharges, according to definitive official figures reported by the medical units of the SS for the period from 2000 to 2016; c) data from the National Population Council (Consejo Nacional de Población, CONAPO) and the 2010 National Population and Housing Census for 1990-2030 population projections in Mexico; and d) data from CONAPO for municipal population projections for 2010-2019.

### Variables

Records with a diagnosis of liver disease that was in accordance with the codes of the 10^th^ Revision of the International Classification of Diseases (ICD-10) were selected. The corresponding figures for the study variables were obtained from the databases by applying the Global Burden of Disease (GBD) classification to analyze deaths and hospital discharges, based on the risk factors for HCC described in the literature.^2^ ^,^ ^6^ ^,^ ^8^ Thus, for chronic viral hepatitis (CVH) B or C, the code was B18; for MLT, C220-C224, C227 and C229; for ALD, K70; and for liver cirrhosis, K74. NASH and MetS were not considered because of limited availability of information.

### Study size, eligibility criteria, calculations and statistical analysis

The sample size depended on inclusion of consecutive cases sampled from the total figures in the databases according to year, after applying the exclusion criteria. The mortality rates were calculated annually from 1990 to 2019, with regard to reports of deaths in municipalities of Hidalgo State (towns and their areas), and were analyzed according to sex and age groups. The mortality rate in Hidalgo was compared with that of other Mexican states. The DGIS records of death due to MLT as the primary cause over the 34-year period from 1985 to 2019 were used to run functional models for data trend projections. These revealed a positive association between time and death due to liver cancer; the actual figures were compared with those projected using the Student t test.

To determine whether the hospital discharges relating to liver cancer were correlated with the hospital discharges relating to pathological conditions that were considered to be causes of HCC (which was our hypothesis), data from all municipalities were studied using the Spearman correlation test. However, the data did not follow normal distribution (P < 0.0001). Since not every municipality had similar hospital discharge relating to liver cancer, that hypothesis was tested again but considering only the municipalities in which the death rate was higher than the mean (5.02) + one standard deviation (5.49). These corresponded to municipalities with at least twice the national death rate due to HCC. These data followed normal distribution (P > 0.05), and the Spearman correlation test was thus applied. Finally, normality was tested using the Shapiro-Wilk test. These analyses were performed using the GraphPad Prism 6.01 software (California, United States).

The inclusion criteria were that these municipalities of Hidalgo State needed to have the following: a) records of MLT as the primary cause of death; and b) records of hospital discharges from the SS of Hidalgo relating to liver diseases. The exclusion criteria were situations of the following: a) death and hospital discharge records that stated a country of residence other than Mexico or reported the country as unspecified; and b) for data analysis at the state level, death and hospital discharge records declaring a state of residence other than Hidalgo or reporting this as unspecified.

Descriptive analysis on the data was carried out by determining the relative weight and mortality rate (national or state), and by numbering the position occupied by each tumor as a cause of death due to malignant neoplasm. Crude or cumulative death rates were calculated to compare the mortality rate in Hidalgo State with the rates in other Mexican states and the MLT mortality rate for the country over the period 1990-2019. Crude or cumulative death rates were estimated to compare the 84 municipalities of Hidalgo State from 1990 to 2019. The 1990-2030 population projections for Mexico compiled by CONAPO were used.

After the municipal mortality rates for liver cancer had been calculated, differential mapping was used to identify regions of Hidalgo with higher liver cancer rates. In addition, the 2010-2019 municipal population projections for Mexico compiled by CONAPO were used.

## RESULTS

The relative weight (%), mortality rates for malignant tumors and their ranks as the primary cause of mortality were calculated for Mexico and Hidalgo based on the death figures for malignant tumors according to the GBD classification (Table 1). MLTs are a public health problem at both the national and the state level, considering that they are the fifth largest cause of death in Mexico and the leading cause in Hidalgo. This state’s liver cancer mortality rate is 6.4 deaths per 100,000 inhabitants, which is 1.1 points greater than the national rate; its relative weight is at least 1.4% higher than that of other tumors in the state and Mexico.

**Table 1.** The six malignant tumors with the highest mortality rates in Mexico and Hidalgo in 2019

Tumor	Deaths in Mexico	Relative weight (%)	Rate	National rank	Deaths in Hidalgo	Relative weight (%)	Rate	State rank
Breast	7,419	8.5	5.9	1	152	7.7	5.0	3
Colorectal	6,978	8.0	5.5	2	142	7.2	4.7	4
Trachea, bronchi and lung	6,774	7.8	5.4	3	112	5.7	3.7	7
Prostate	6,760	7.8	5.34	4	160	8.1	5.2	2
Liver	6,727	7.8	5.3	5	194	9.9	6.4	1
Stomach	6,327	7.3	5.0	6	141	7.2	4.6	5
Total	40,985	47.2	32.44		901	45.8	29.6	

General Directorate of Health Information (DGIS), Institute of Statistics, Geography and Informatics (INEGI), Ministry of Health (Secretaría de Salud, SS) of Mexico and National Population Council (Consejo Nacional de Población, CONAPO). Definitive official death figures in 2019. Rates are calculated per 100,000 inhabitants within the total population for the period and were consulted in September 2021.

Table 2 shows the ten states of Mexico that had the highest MLT mortality rates over the period 1990-2019. The increase in the ranking of the Hidalgo figures is evident: from the 21^st^ place in 1990 to ninth place in 2019. Hidalgo occupied the sixth place in 2005; since then, it has always been among the top ten. Hidalgo occupies the ninth place in the national ranking of liver cancer deaths considering the cumulative death rate of 2010-2019 as the period analyzed for mortality. Furthermore, the ECI index in 2021 for each state is presented to contextualize their economic development. As observed, the situation of the financial resources of most of these states has the implication that their ability to improve their healthcare services and prevention programs may be limited.

**Table 2.** The current ten states of Mexico with the highest mortality rates for malignant liver tumors, 1990-2019

State	1990	^*R*^	1995	^*R*^	2000	^*R*^	2005	^*R*^	2010	^*R*^	2015	^*R*^	2019	^*R*^	ECI 2021
Veracruz	4.34	^*4*^	5.85	^*3*^	6.73	^*3*^	8.29	^*1*^	8.56	^*1*^	10.08	^*1*^	9.13	^*1*^	-0.95
Oaxaca	2.47	^*25*^	3.65	^*15*^	4.36	^*9*^	4.85	^*9*^	5.82	^*6*^	6.68	^*4*^	7.96	^*2*^	-1.97
Yucatan	6.23	^*1*^	8.57	^*1*^	7.26	^*1*^	6.49	^*3*^	6.36	^*3*^	7.13	^*3*^	7.97	^*3*^	0.06
Tabasco	3.85	^*12*^	4.52	^*7*^	4.25	^*11*^	4.53	^*12*^	5.15	^*11*^	5.91	^*11*^	7.00	^*4*^	-0.75
Chiapas	3.59	^*14*^	4.42	^*8*^	4.54	^*8*^	4.99	^*8*^	5.79	^*7*^	6.43	^*7*^	6.92	^*5*^	-1.77
Campeche	2.35	^*26*^	4.92	^*5*^	4.14	^*14*^	4.43	^*13*^	4.42	^*16*^	7.16	^*2*^	6.91	^*6*^	-0.43
San Luis Potosi	3.13	^*20*^	3.84	^*13*^	4.81	^*7*^	5.53	^*5*^	6.15	^*4*^	6.14	^*9*^	6.75	^*7*^	0.64
Mexico City	3.59	^*15*^	4.53	^*6*^	5.02	^*6*^	5.72	^*4*^	5.94	^*5*^	6.54	^*5*^	6.65	^*8*^	0.95
Hidalgo	3.08	^*21*^	3.64	^*16*^	3.1	^*27*^	5.51	^*6*^	5.39	^*9*^	6.43	^*8*^	6.36	^*9*^	-0.68
Tamaulipas	5.5	^*3*^	5.63	^*4*^	5.52	^*4*^	5.27	^*7*^	7.32	^*2*^	6.43	^*6*^	5.83	^*10*^	0.81

ECI = economic complexity index 2021.^11^

General Directorate of Health Information (DGIS), Institute of Statistics, Geography and Informatics (INEGI), Ministry of Health (Secretaría de Salud, SS) of Mexico and National Population Council (Consejo Nacional de Población, CONAPO). R: The annual national rank of the state in mortality rates for malignant liver tumors calculated per 100,000 inhabitants. Definitive official death figures (1990-2019) were consulted in September 2021.

**Figure 1.**
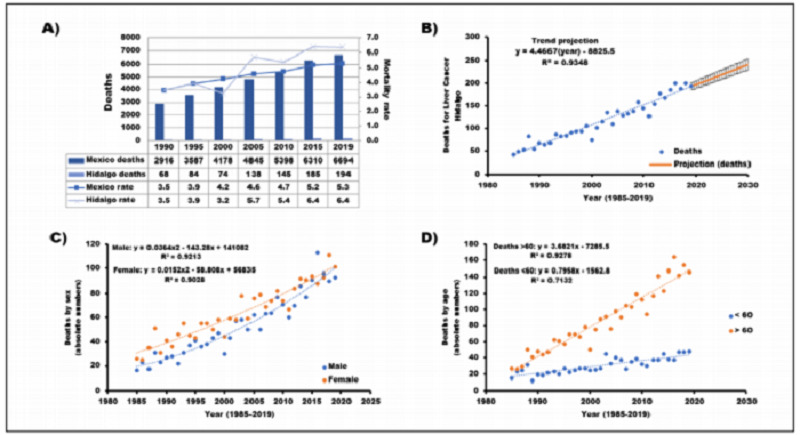
A) Comparison of mortality rates for malignant liver tumors between Hidalgo State and Mexico, 1990-2019. B) Deaths due to liver cancer in the Hidalgo State population, 1985-2019, and 2018-2030 projections. Error bars represent the 95% confidence interval. C) Quadratic trend according to sex of liver cancer deaths, 1985-2019. D) Linear trend according to age group (< 60 years or > 60 years) of liver cancer deaths, 1985-2019.

The MLT mortality rate was 31.43% higher than the national rate between 1990 and 2019 (151.42% for Mexico and 182.85% for Hidalgo). Since 2005, the mortality rates in Hidalgo have always been higher than national rates (Figure 1A). The death records comprising the period from 1985 to 2019 formed the basis to construct a functional linear model of data trend (Figure 1B), which showed a positive association between time and liver cancer death (y = 4.4667x - 8825.5; R^2^ = 0.935), as well as a noticeable increase in the number of deaths due to this cause over time. This relationship was corroborated by comparing real and theoretical figures (t_64_ = 0.0002; P = 0.99), which were statistically similar. This model projected 237 deaths (95% confidence interval, CI, 223 to 251) due to MLT by 2030, in contrast to 194 deaths recorded in 2019 (122.16% increase). Figure 1C depicts an increasing trend in deaths for both men (y = 0.0364x^2^ - 143.28x + 141082; R^2^ = 0.921) and women (y = 0.0152x^2^ - 58.808x + 56835; R^2^ = 0.903) from 1985-2019 through a quadratic trend model. A larger number of deaths among women can be seen, although more men died in some years; however, these figures are similar when the data are compared according to mortality rates. The distribution according to age group with liver cancer as the cause of death (1985-2019) showed a linear trend (< 60 years old, y = 0.7958x - 1562.8, R^2^ = 0.713; > 60 years old, y = 3.6821x - 7285.5, R^2^ = 0.928), in which a greater increase over time was observed for the group aged > 60 years (Figure 1D).

Adjusted liver cancer mortality rates according to sex and age in 2010-2019 were calculated to compare Mexico with Hidalgo, but only the 2019 figures are presented because the trend over time was similar. For individuals > 60 years old in Mexico (rate 37.8 per 100,000 inhabitants), the rate for females was 34.2 and for males, 41.9; in Hidalgo (rate 42.1), the rate for females was 38.0 and for males, 47.0. For individuals < 60 years old in Mexico (rate 1.2), the rate for females was 1.1 and for males, 1.2; in Hidalgo (rate 1.8), the rate for females was 2.2 and for males, 1.4. Thus, Hidalgo had higher mortality rates for both sexes and both age groups than those of Mexico. Standardized rates were greater for males and > 60 years in both Hidalgo and Mexico, except for females < 60 in Hidalgo.

**Figure 2,:**
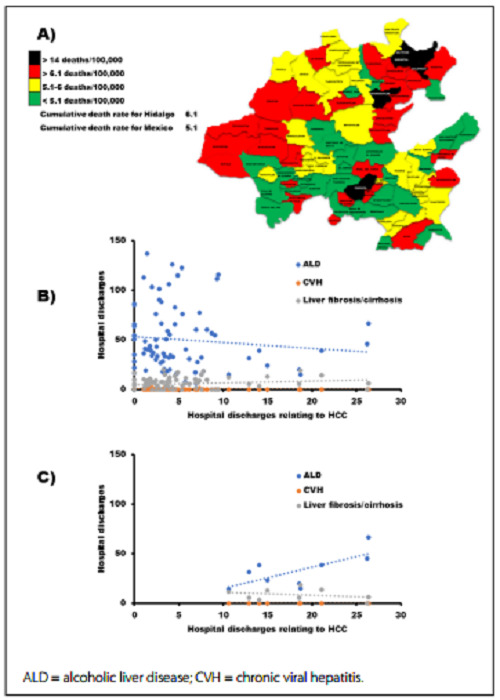
A) Map of distribution of deaths due to hepatocellular carcinoma (HCC) according to municipality and cumulative death rates in Hidalgo State, using data from the General Directorate of Health Information (DGIS) and Ministry of Health (Secretaría de Salud, SS) of Hidalgo, 2010-2019. B) Correlation between hospital discharges relating to HCC and hospital discharges relating to risk factors for this disease in the municipalities of Hidalgo State. C) Correlation between hospital discharges relating to HCC and hospital discharges relating to risk factors for this disease in the nine municipalities of Hidalgo with the highest HCC death rates.

Given the high prevalence of MLT in the state, municipal disaggregation was a necessity in order to analyze their death rates over the period 2010-2019. Figure 2A shows a map of Hidalgo State in which the municipalities are color-coded as follows: municipalities with a liver cancer death rate lower than the national rate are shown in green; those with a death rate between 5.1 and 6.1, which are the national and state cumulative rates respectively, are shown in yellow; those with a rate higher than the state rate are shown in red, and those with the highest rates (twice the state rate or more) are shown in black. Huejutla was the municipality with the largest number of deaths (187) due to MLT and had the third highest mortality rate. Jaltocan ranked first in mortality rate despite its small population and just 20 deaths. These two municipalities are considered socially marginalized; surprisingly, Pachuca, the state capital municipality, had a mortality rate of 14 with 141 deaths. The population > 60 years exhibited the largest number of deaths; however, there were reports of deaths among young patients (23-39 years of age) in critical municipalities. In the five municipalities with the highest mortality rate, the percentage of death among women was slightly higher than that among men (52.8% versus 47.2%), although some municipalities registered more male deaths.

The hospital discharge rates were calculated considering the SS records over the period 2010-2019 relating to patients either attended in Hidalgo or from Hidalgo attended in other states, who were diagnosed with CVH, ALD, liver fibrosis/cirrhosis or hepatic cancer. Table 3 shows the 15 municipalities in Hidalgo with the highest hospital discharge rates relating to MLT, according to risk factor for the disease during that period. Figures for the hospital discharge rates of these municipalities can be compared with the state figures.

**Table 3.** The fifteen municipalities in Hidalgo State with the highest hospital discharge rates relating to malignant liver tumors according to risk factors for this disease, 2010-2019

Municipality	Liver cancer	CVH	ALD	Liver fibrosis/cirrhosis
Eloxochitlán	176.4	0.0	52.9	0.0
Pisafores	62.8	0.0	21.8	10.9
Xochicoatlán	40.5	0.0	8.1	8.1
Xochiatipan	30.1	0.0	12.5	10.0
Agua Blanca de Iturbide	20.1	0.0	48.2	4.0
Jacala de Ledezma	19.0	0.0	61.0	9.5
San Agustín Metzquititlán	17.1	0.0	17.1	0.0
Tianguistengo	16.7	0.0	22.3	16.7
Huazalingo	15.0	0.0	5.0	10.0
Tepetitlán	14.7	0.0	33.0	0.0
Nicolás Flores	13.7	0.0	68.3	0.0
Huichapan	13.6	0.5	70.7	1.1
Tlahuelilpan	12.6	0.0	28.7	9.0
Pacula	11.1	0.0	55.5	0.0
Omitlán de Juárez	10.7	0.0	24.9	0.0
Unspecified	10.5	5.3	982.3	124.3
Hidalgo’s hospital discharge rate	0.9	0.1	27.4	4.4

ALD = alcoholic liver disease; CVH: chronic viral hepatitis.

General Directorate of Health Information (DGIS), Institute of Statistics, Geography, and Informatics (INEGI), Ministry of Health (Secretaría de Salud, SS), and National Population Council (Consejo Nacional de Población CONAPO). Definitive official hospital discharge rates calculated per 10,000 inhabitants, 2010-2019. Consulted in September 2021.

The relationship between hospital discharge rates relating to HCC and hospital discharge rates relating to ALD, CVH and fibrosis/cirrhosis as the leading risk factor for the disease in Hidalgo (2010-2019) was studied. The correlation analysis did not show any significant difference between hospital discharges relating to HCC and hospital discharges relating to ADL (ρ = -0.13, P > 0.05), CVH (ρ = -0.19, P > 0.05) or fibrosis/cirrhosis (ρ = 0.16, P > 0.05) (Figure 2B). Nevertheless, among the municipalities with the highest HCC death rates, only nine with a mean hospital discharge rate for HCC + one standard deviation were considered for calculating Pearson’s coefficient. This analysis revealed that there was a significant positive correlation between HCC and ALD (R^2^ = 0.49, P < 0.05) in these critical municipalities (Figure 2C).

## DISCUSSION

In Mexico (ECI index of 1.31 in 2020 and GINI index of 0.45 in 2018), liver cancer is among the five leading types of malignant tumor causing death. This is consistent with the third place that was identified in a review of official death certificates in Mexico over the period 2000-2006, in which a national increase in HCC deaths of 14% was found (4.16 deaths in 2000 versus 4.74 in 2005, both per 100,000 inhabitants); and with the ranking of liver cancer as the third largest cause of mortality due to cancer in GLOBOCAN 2020.^7^ ^,^ ^12^ The trend projections obtained for HCC as a mortality cause are a helpful tool for proposing the strengthening of healthcare strategies to diminish liver cancer cases in Mexico and Hidalgo, since no up-to-date or previous state projections were found.^9^ Cancer is a growing health problem, just like other non-communicable diseases, and reducing deaths caused by such diseases is a goal set by the World Health Organization (WHO).^13^ Similar countries in Latin America, such as Argentina (-0.29 ECI, 0.42 GINI), Brazil (0.10 ECI, 0.53 GINI) and Colombia (0.09 ECI, 0.51 GINI), have lower mortality rates for liver cancer, which was ranked between sixth and tenth place in 2020.^12^

MLTs are the leading cause of death in Hidalgo; identification and characterization of critical municipalities provide better understanding of the current situation of this disease in the state. In the population studied, the highest mortality rates were observed in the group aged > 60 years, which agrees with earlier data for Mexico.^8^ ^,^ ^10^ In addition, both sexes seemed to suffer equally from HCC in 2014,^8^ although most authors have reported higher mortality rates for men in Mexico^5^ ^,^ ^6^ and Latin America.^2^ In the present study in 2019, a higher death rate among women aged < 60 years was found in Hidalgo, although men were more affected than women in the three municipalities with the highest HCC death figures. Epidemiological reports have shown an equal ratio of male/female mortality due to HCC in countries like Mexico; however, current trends indicate a rise in the number of female deaths, such that in Mexico liver cancer was the third largest cause of death among women and the fifth among men in 2020.^9^ ^,^ ^12^ ^,^ ^14^ Conversely, the estimated death rates due to liver cancer in Argentina, Brazil and Colombia for both sexes ranked sixth to tenth among cancers in 2020.^12^

Scientific evidence confirms that ALD, CVH and fibrosis/cirrhosis are the major risk factors for developing liver cancer.^1^ ^,^ ^2^ ^,^ ^3^ Several studies carried out in South America have reported that HCV infection is the most frequent risk factor, but also that HCV/HBV coinfection, alcoholic cirrhosis and non-alcoholic fatty liver disease (NAFLD) are also widespread risk factors.^4^ Indeed, the Mexican population has been found to be more susceptible to ALD because of genetic and environmental factors.^15^

In addition to the mortality data, our analysis on hospital discharges among Hidalgo inhabitants provided a gross indicator of morbidity. Hospital discharges can represent either demand for or provision of healthcare services, thus providing a valuable tool for identifying the morbidity profile. Moreover, hospital discharges indicate the level of hospital services for resolving the needs of patients.^16^ For the present study, hospital discharges in which the municipality of residence was not specified were eliminated from the results, given that the objective was to analyze municipalities; otherwise, precise information might improve the municipal analysis.

A significant increase in hospital discharges among cirrhosis patients was observed in Hidalgo from 2010 to 2019. Other authors have also reported high incidence and prevalence of cirrhosis over a similar period, which was related to high alcohol consumption and viral infections.^9^ ^,^ ^17^ Hospital discharges relating to HCV also increased in this state, since chronic HCV infection has emerged as a health problem in Mexico^18^ ^,^ ^19^ and Latin America.^3^ ^,^ ^4^ ALD is the most important cause of chronic liver disease and accounts for one-third of all HCC cases globally.^20^ Furthermore, the increase in alcohol consumption in the young Mexican population is alarming, while demand for its medical treatment is diminishing.^21^ ^,^ ^22^ ^,^ ^23^ Moreover, there is an increasing trend of injectable drug abuse.^19^ ^,^ ^24^ This information may explain the increase in the rate of hospital discharges relating to ALD in Hidalgo. These data support the existence of a correlation between HCC in Hidalgo and the risk factors of ALD, CVH and cirrhosis.

Studies on HCC epidemiology in Argentina, Brazil and Chile, among other countries, have pointed out the importance of NAFLD/NASH as a new leading cause of liver cancer.^4^ In Mexico, specific information about HCC caused by NAFLD/NASH is scarce.^7^ ^,^ ^8^ ^,^ ^9^ The lack of data on the prevalences of NASH, MetS and alcoholism in the municipalities of Hidalgo hinders implementation of effective public health strategies and policies. Therefore, enhanced prevention programs to decrease the acquisition of modifiable risk factors are essential, including hepatitis B vaccination. Likewise, there is a need for programs promoting healthy nutrition and safe sex and programs aimed at stopping injection drug use to prevent HCV infection and aimed at reducing alcohol consumption. Moreover, early diagnosis and treatment adherence are needed.^2^ ^,^ ^8^ ^,^ ^19^ ^,^ ^23^ ^,^ ^24^

The municipal cumulative MLT mortality rates revealed the presence of a critical zone in the north of Hidalgo, with a rate that was almost three times higher than the national rate. Indeed, some municipalities with high hospital discharge rates regarding HCC also show many hospital discharges relating to ALD and cirrhosis. Therefore, a significant correlation between HCC and ALD was found in these municipalities. Notably, in Hidalgo State, 43.8% of the population lives in poverty and 6.1% under conditions of extreme poverty.^11^ ^,^ ^25^ The northern region exhibits high poverty indicators, dispersion of small communities, underserved populations and indigenous residents, according to DGIS 2019 data. In addition, it has a high illiteracy rate (6% to 20% in 2020) and high social inequality according to the GINI index (0.435-0.505 in 2015).^11^

Unexpectedly, the municipality of Pachuca, the state capital of Hidalgo, had a high mortality rate for liver cancer and was ranked third. Pachuca had a GINI index of 0.397 in 2015 and an illiteracy rate of 1.59% in 2020. Use of alcohol and illegal drugs is increasing in Pachuca, although 54.8% of the Hidalgo population has been exposed to prevention programs.^22^ Hence, these municipalities require a profound status analysis and regional strategies to improve future public health policies.

These findings suggest that national or state prevention programs aimed at reducing alcohol and drug consumption or at providing care for underserved communities have not significantly improved the social and health conditions in those municipalities. This may be considered to be a social failure despite the many governmental strategies that have been implemented to improve Mexico’s poverty and social lag indicators.^22^ ^,^ ^23^ ^,^ ^24^ ^,^ ^25^ Consequently, the prevalence of non-communicable diseases is high among marginalized communities. Moreover, these communities are exposed to unhealthy environments, and the socioeconomic inequalities and exclusion from formal labor markets to which these communities are exposed in Mexico and Latin America restrict their access to healthcare services.^26^ Although local or regionalized public health policies are often overdue, application of such policies is required urgently because they can have profound positive effects on community health and can ameliorate health disparities, lessen administrative paperwork within healthcare systems and diminish governmental dysfunction.^27^ Two areas of opportunity and challenges are recognized: implementation of a population-based cancer registry with reliable data; and creation of a national cancer plan to guide control programs and strategies.^28^ ^,^ ^29^ Unfortunately, local or regional healthcare policy strategies depend on government budgets at the state or national level, but community and municipal populations cannot quickly manage these resources and demand their uncorrupted use.

## CONCLUSION

This study provided a detailed epidemiological view of the status of liver cancer in Hidalgo State through projections, trends, and cause analysis. This information may serve as a helpful example with regard to identifying a local health issue that requires establishment of preventive actions to diminish recognized and correlated risk factors, particularly in marginalized municipalities. In this regard, increased recording and surveillance of NASH and MetS are mandatory since these are currently the second most common risk factors for HCC. Lastly, there is an urgent need for effective regional health policies and strategies in Hidalgo State, Mexico, and throughout Latin America and in other countries with similar epidemiological and socioeconomic conditions, in order to prevent the expansion of liver cancer to populations that are more vulnerable to alcoholism and other risk factors for liver cancer.
